# An experimental comparison and user evaluation of three different dried plasma products

**DOI:** 10.1111/vox.13798

**Published:** 2025-01-27

**Authors:** Kristina Ehn, Gabriel Skallsjö, Birgitta Romlin, Göran Sandström, Per Sandgren, Agneta Wikman

**Affiliations:** ^1^ Clinical Immunology and Transfusion Medicine Karolinska University Hospital Stockholm Sweden; ^2^ Department of Center for Hematology and Regenerative Medicine (HERM) Karolinska Institutet Stockholm Sweden; ^3^ Department of Anaesthesiology and Intensive Care, Institute of Clinical Sciences, Sahlgrenska Academy University of Gothenburg Gothenburg Sweden; ^4^ Department of Anaesthesiology Södra Älvsborgs Sjukhus Borås Sweden; ^5^ Helicopter Emergency Medical Service Västra Götalandsregionen Gothenburg Sweden; ^6^ Institute of Clinical Sciences, Department of Pediatric Anesthesia and Intensive Care, Sahlgrenska Academy University of Gothenburg Gothenburg Sweden; ^7^ Department of War Studies Swedish Defense University Stockholm Sweden

**Keywords:** dried plasma, haemorrhage, major trauma, plasma transfusion, pre‐hospital

## Abstract

**Background and Objectives:**

Access to blood components in pre‐hospital bleeding resuscitation is challenging. Dried plasma is a logistically superior alternative, and new products are emerging. Therefore, we aimed to evaluate laboratory and practical differences in three differently produced dried plasma products.

**Materials and Methods:**

Single‐donor lyophilized LyoPlas®, pooled‐donor, lyophilized and pathogen‐reduced OctaplasLG Powder®, and single‐donor sprayed‐dried FrontlineODP™ along with fresh plasma (in‐house, pre‐FrontlineODP and OctaplasLG) as controls were analysed (*n* = 8). Laboratory tests included measurements of various coagulation factors and thromboelastography. The practical evaluation of the dried plasma products included preparation time, time to dissolve the dried plasma and total time, together with subjective opinions from eight clinical users.

**Results:**

The coagulation factor content was within human reference ranges for all dried plasma, with approximately 10%–20% loss compared with fresh plasma. More variations were observed in the single‐donor products compared with the pooled products. Clot formation analysed by thromboelastography showed normal graphs. Reconstitution time was similar, ranging from on average 7–9 min. In the user evaluation, the reconstitution time and the possibility of using a plastic bag for the transfusion were emphasized as important, the latter fulfilled by two of the products.

**Conclusion:**

The study supports that dried plasma may be produced with lyophilization or spray‐drying technique, as well as with the addition of pathogen reduction, with preserved coagulation capability. The products were reconstituted in acceptable time and deemed feasible for pre‐hospital use by eighth test users.

**Highlights**

- The dried plasma products, manufactured in three different ways, exhibited a comparable concentration of coagulation factors and normal clot formation when analysed by thromboelastography.
- The three dried plasma products could all be reconstituted in less than 10 min, which could potentially be reduced to less than 5 min with practice.
- Quick reconstitution and the final product being packaged in plastic bags were emphasized as important by potential dried plasma users.

## INTRODUCTION

Plasma transfusion in pre‐hospital care and in military battlefields may potentially increase survival and minimize downstream complications related to haemorrhage [1]. However, direct access to blood components in such settings is logistically challenging. Freeze‐ or sprayed‐dried plasma (DP) is logistically superior to fresh frozen plasma (FFP) as it can be stored for long periods at ambient temperature and is thus suitable for on‐site treatment [2, 3].

DP has been used since the Second World War, but usage was stopped in the 1960s due to pathogen transmission. Later, in 1990s, improved strategies in donor screening and testing along with new possibilities to pathogen‐reduce blood components re‐introduced the usage of DP [4, 5, 6]. The most common drying technology is lyophilization, which takes several days of freezing under a vacuum to reduce the water content to about 1%–2% [7]. Another alternative is spray‐drying which can be accomplished within 25 min, as liquid plasma droplets are atomized in a chamber [8]. After production, the shelf life of DP is 18–24 months, with the potential for further extension of the storage time [9].

Several in vitro studies have shown that DP contains similar amounts of coagulation proteins to fresh plasma, although greater reductions in von Willebrand (vWF) and labile factors V and VIII were observed [6, 8, 10, 11, 12]. Sprayed‐DP has been shown to further reduce the vWF levels, but newer optimized methods have counteracted this loss [8, 13]. However, there is little or no evidence about the specific plasma protein activity level required to obtain the clinical efficacy of plasma transfusion. Therefore, minor reductions in coagulation factors may be negligible as clinical experience has shown promising results for the stabilization of bleeding patients [5, 14, 15].

Until now, there have only been two products available in Europe and the United States, a single‐donor non‐pathogen‐reduced product from the German Red Cross and a pooled pathogen‐reduced product from the French Armed Forces Blood Transfusion Centre (CTSA). Today new products are emerging. OctaplasLG Powder (Octapharma® Nordic AB, Stockholm, Sweden) is a recently approved pharmaceutical product available in the market. FrontlineODP (Velico® Medical, MA, USA) has developed a system for in‐house production of spray‐DP, under approval in United States and Europe [16].

Due to an increased interest in DP and new production lines in blood establishments or products from pharmaceutical companies offered in the near future, studies comparing different plasma alternatives are called for. Therefore, our first aim was to compare the in vitro quality of three differently produced DP: LyoPlas N‐w, OctaplasLG Powder and FrontlineODP. Second, we aimed to evaluate the practical aspects of each DP product.

## MATERIALS AND METHODS

### Ethics statement

The study was conducted in accordance with the Declaration of Helsinki. The Swedish Regional Ethics Review Boards advisory had no ethical objections regarding this research project (Dnr. 2021‐01321).

### Study protocol

**FIGURE 1:**
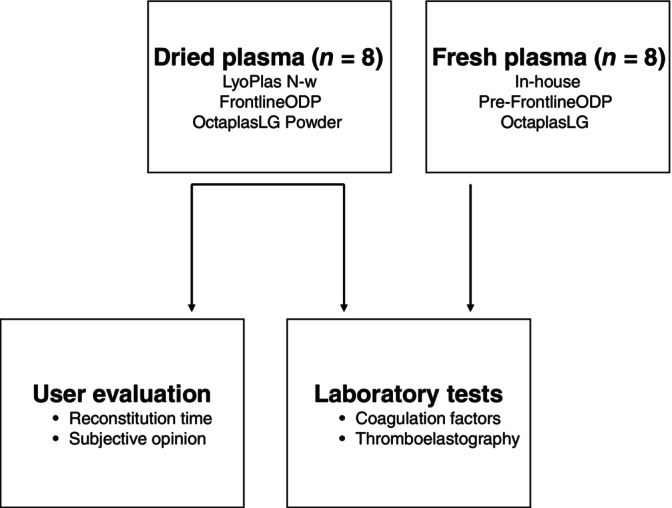
Study design. The figure displays the study design evaluating dried plasma focusing on laboratory tests and practical aspects of the plasma reconstitution, in parallel.

The experimental study consists of in vitro analyses to determine coagulation qualities, and a practical evaluation by medical personnel of various backgrounds (Figure 1). Eight units of each DP product were evaluated, and the included products were LyoPlas N‐w (Red Cross, Germany), OctaplasLG Powder (Octapharma® Nordic AB) and FrontlineODP™ (Velico® Medical). The laboratory tests were analysed in parallel with three different fresh plasma products (eight units of each) as controls, FFP produced at Karolinska University Hospital, OctaplasLG (Octapharma® Nordic AB) and frozen pre‐samples from each unit of FrontlineODP™ (Velico® Medical).

### Characteristics of the plasma products

LyoPlas N‐w is produced at the Red Cross in Germany. It is dried through lyophilization and is single unit, blood group‐specific and non‐pathogen‐reduced. The product is an approved pharmaceutical product in European countries. It consists of a packaging with a glass bottle with freeze‐DP, a plastic bag with 200 mL sterile water and a transfer set with a spike and lock connector. The analysed LyoPlas units were of blood group AB. The storage time from production is 18 months according to the manufacturer [9].

OctaplasLG Powder is a lyophilized, pooled pathogen‐reduced pharmaceutical product, recently approved and released. The production is based on the same production line as OctaplasLG with large pools of 630–1520 donors and treated with solvent detergent technology [6]. The packaging is a glass bottle with freeze‐DP and a plastic bag with 200 mL of sterile water, including a transfer set with spike and needle. The product is blood group‐specific and the analysed plasma units were blood group AB. The storage time is 24 months according to the manufacturer.

FrontlineODP™ is a product intended for production in the blood bank. Installation of the FrontlineODP™ system is required for the spray‐drying process, but in this evaluation, the system was not installed. The spray‐dried product is prepared from a single unit of non‐pathogen‐reduced fresh plasma, transferred into a plastic bag and delivered with 200 mL of sterile water in a plastic bag along with a double‐ended spike transfer set. In this study, six blood group A and two blood group B units were examined. The storage time is currently validated up to 24 months.

As controls, three different fresh plasma products were analysed. FFP produced at Karolinska University Hospital, of blood group A (*n* = 4) and AB (*n* = 4) and frozen within 20 h, OctaplasLG (Octapharma® Nordic AB) blood group AB (*n* = 4) and A (*n* = 4), from two different batches and frozen pre‐samples from each unit of FrontlineODP™ (Velico® Medical). The different fresh plasmas were stored frozen at <−35°C until the day of analysis, and the DP and sterile water were stored at 2–8°C. All DP were analysed within expiry.

### Concentration of coagulation factors

Analysis of coagulation factor content in the plasma products was performed on STA Compact Max® 3 (Stago, France) according to the manufacturer's recommendations and instructions. The following analyses were performed: Fibrinogen (g/L) STA‐Liquid Fib (Stago), factor FVIII (UI/mL) STA‐Deficient VIII (Stago), activated partial thromboplastin time (aPTT) (s) STA‐PTTA (Stago), vWF antigen (UI/mL) LIA test vWF:Ag (Stago), Owren prothrombin time (PT) (INR) STA‐SPA+ (Stago), Protein C (UI/mL) Stachrom Protein C (Stago), antithrombin III (IU/mL) STA‐Stachrom‐AT‐III (Stago) and FXIII (%) K‐Assay FXIII (Kamiya Biomedical Company, Seattle, WA, USA). The DP was analysed within an hour after reconstitution while frozen in‐house plasma, Pre‐FrontlineODP, were thawed <10 min on a heatblock (VWR International AB, Sweden) and OctaplasLG <20 min using Barkey Plasmatherm DTM (Barkey, Germany), directly upon analysis.

### Thromboelastography

The clot‐forming ability of plasma was analysed on TEG® 6S (Haemonetics® Coperations, Boston, MA, USA) using a four‐channel cartridge Global Haemostasis (Haemonetics® Coperations). Reconstituted DP was analysed within 4 h, and the fresh plasma was analysed within 4 h from thawing. Each plasma sample was mixed at 1:1:1 with fresh platelets (day 1 from production) and stored red blood cells (<14 days) of blood group O+. Erythrocyte haematocrit and platelet count were analysed with cell counter SweLab Alfa (Boule, Sweden) to ensure similar proportions. Rapid thromboelastography (TEG) and TEG functional fibrinogen were chosen as the readout with parameters; clotting time® corresponding to a clot amplitude of 2 mm, clot kinetics (*K*) measuring the time from 2 to 20 mm clot, clot strengthening rate (*α*, angle) reflects the velocity of clot strength generation, and amplitude 10 min (A10) equals the clot firmness 10 min after *R*, representing the platelet‐fibrin clot strength [17].

### User evaluation

Eight potential DP product users (two laboratory staff, two anaesthesiologists, two anaesthesia nurses and two combat medics) representing pre‐hospital, in‐hospital and military care performed the reconstitution and evaluated the products. Written instructions were given before reconstitution for each brand of DP; additionally, a short video instruction was mandatory to watch for LyoPlas N‐w and FrontlineODP™. Time measurements were documented, including (1) preparation time: time to connect and transfer the sterile water; (2) time to dissolve: time for the DP to dissolve, starting when all sterile water was transferred; and (3) total time: from start (open packaging) until the user determined the plasma to be ready for transfusion. They were also asked to give their subjective view of the products, including the packages. Foam was assessed from 0 = no foam to 4 = excessive foam. Subjective opinions were documented in the protocol throughout the procedure.

### Statistics

The statistical data were analysed using GraphPad Prism 10.0.3 (GraphPad Software Inc., CA, USA). The mean values with standard deviations are presented along with figures displaying boxes and whiskers representing the median with minimum and maximum values. To compare the different dried and fresh plasma one‐way analysis of variance with Tukey's multiple comparison or Kruskal–Wallis test with Dunn's multiple comparison was used depending on the assumption of normal distribution within each group determined by the Shapiro–Wilk test. The statistical significance was set to *p* < 0.05.

## RESULTS

### Coagulation factor content

**FIGURE 2:**
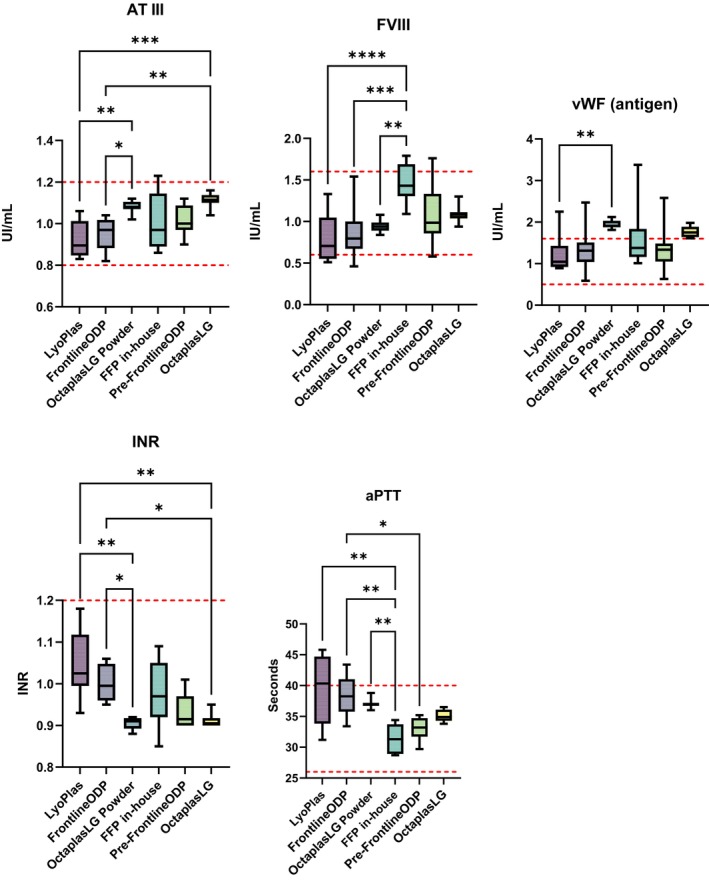
Coagulation factors in plasma. The figure presents graphs of the coagulation parameters with significant variations. Each group consists of eight samples displayed with boxes and whiskers defining the median with min and max values. Red dotted line represents human reference interval. Significant differences according to multiple comparison test (Turkey's or Dunn's) are defined as *p < 0.05, **p < 0.01, ***p < 0.001, ****p < 0.0001. aPTT, activated partial thromboplastin time; AT III, antithrombin III; FFP, fresh frozen plasma; vWF, von Willebrand factor.

The mean value of each analysed coagulation factor was within normal human reference intervals in the DP products, as well as the fresh plasma. Variations in concentrations were greater in the single‐donor products compared with the pooled products from Octapharma. The concentration of fibrinogen, protein C and FXIII was stable in the DP (Table 1). The remaining coagulation parameters displayed reductions in concentration, compared with fresh plasma (Figure 2). Factor VIII content was highest in the in‐house fresh plasma group, and significantly higher than in all DP products. Moreover, aPTT was significantly shorter in the in‐house fresh plasma compared with all DP products (Figure 2).

**TABLE 1:** Concentration of coagulation factors in dried and fresh plasma (mean ± SD).

Ref. values	LyoPlas, *n* = 8 (AB)	FrontlineODP, *n* = 6 (A), *n* = 2 (B)	OctaplasLG Powder, *n* = 8 (AB)	FFP in‐house, *n* = 4 (AB), *n* = 4 (A)	Pre‐FrontlineODP, *n* = 6 (A), *n* = 2 (B)	OctaplasLG, *n* = 4 (AB), *n* = 4 (A)	*p*‐Value
Fibrinogen, 2–4 g/L	2.72 ± 0.55	2.69 ± 0.55	3.11 ± 0.06	2.97 ± 0.57	2.80 ± 0.58	3.00 ± 0.13	ns
FVIII, 0.6–1.5 IU/mL	0.80 ± 0.29^a^	0.87 ± 0.32^b^	0.94 ± 0.07^c^	1.45 ± 0.24^a^, ^b^, ^c^	1.08 ± 0.36	1.09 ± 0.10	0.0001
Protein C, 0.7–1.3 IU/mL	0.90 ± 0.16	0.89 ± 0.16	1.07 ± 0.04	1.02 ± 0.18	0.92 ± 0.17	1.08 ± 0.03	ns
AT III, 0.8–1.2 IU/mL	0.92 ± 0.09^d^, ^e^	0.95 ± 0.08^f^, ^g^	1.08 ± 0.03^d^, ^f^	1.02 ± 0.14	1.02 ± 0.07	1.11 ± 0.04^e^, ^g^	<0.001
vWF, 0.5–1.6 IU/mL	1.24 ± 0.46^d^	1.34 ± 0.55	1.94 ± 0.10^d^	1.62 ± 0.77	1.37 ± 0.57	1.77 ± 0.14	<0.01
FXIII, 59%–181%	124.6 ± 32.7	135.0 ± 35.8	109.6 ± 10.6	118.5 ± 28.0	134.3 ± 23.8	103.3 ± 7.7	ns
INR, <1.2	1.05 ± 0.08^d^, ^e^	1.00 ± 0.04^f^, ^g^	0.90 ± 0.01^d^, ^f^	0.98 ± 0.08	0.93 ± 0.04	0.91 ± 0.02^e^, ^g^	<0.0001
aPTT, 26–40 s	39.39 ± 5.55^a^	38.49 ± 3.24^b^, ^h^	37.09 ± 0.80^c^	31.39 ± 2.55^a^, ^b^, ^c^	33.04 ± 1.86^h^	35.06 ± 0.96	<0.0001

*Note*: *p*‐Value was derived from one‐way ANOVA or Kruskal–Wallis test depending on normality.

Abbreviations: ANOVA, analysis of variance; aPTT, activated partial thromboplastin time; AT III, antithrombin III; FFP, fresh frozen plasma; ns, non‐significant; vWF, von Willebrand factor.

^a^ FFP in‐house versus LyoPlas. FVIII *p* < 0.0001 (Tukey's); aPTT *p* < 0.01 (Dunn's).

^b^ FFP in‐house versus FrontlineODP. FVIII *p* < 0.001 (Tukey's); aPTT *p* < 0.01 (Dunn's).

^c^ FFP in‐house versus OctaplasLG Powder. FVIII *p* < 0.01 (Tukey's); aPTT *p* < 0.01 (Dunn's).

^d^ LyoPlas versus OctaplasLG Powder. AT III *p* < 0.01 (Tukey's); vWF *p* < 0.01 (Dunn's); INR *p* < 0.01 (Dunn's).

^e^ LyoPlas versus OctaplasLG. AT III *p* < 0.001 (Tukey's); INR *p* < 0.01 (Dunn's).

^f^ FrontlineODP versus OctaplasLG Powder. AT III *p* < 0.05 (Tukey's); INR *p* < 0.05 (Dunn's).

^g^ FrontlineODP versus OctaplasLG. AT III *p* < 0.01 (Tukey's); INR *p* < 0.05 (Dunn's).

^h^ FrontlineODP versus Pre‐FrontlineODP. aPTT *p* < 0.05 (Dunn's).

### Thromboelastography

The TEG analysis required the plasma products to be mixed (1:1:1) with erythrocytes and platelets. After mixing, the haematocrit (%) (FFP: 23 ± 2, LyoPlas: 23 ± 2, Pre‐Frontline: 23 ± 2, FrontlineODP: 24 ± 3, Octaplas: 24 ± 3, Octaplas Powder: 25 ± 2) and platelet count (platelets 10^9^/L) (FFP: 342 ± 38, LyoPlas: 342 ± 35, Pre‐Frontline: 334 ± 34, FrontlineODP: 338 ± 29, Octaplas: 331 ± 36, Octaplas Powder: 345 ± 39) showed no significant difference. The assays citrated rapid TEG (CRT) and citrated functional fibrinogen (CFF) displayed no significant differences between the DP products. Minor changes compared with fresh products were observed. LyoPlas showed a lower A10 amplitude in the CRT channel compared with FFP in‐house (*p* = 0.003) and pre‐FrontlineODP (*p* = 0.016). The angle of FrontlineODP was decreased compared with FFP in‐house, *p* = 0.043 (Table 2).

**TABLE 2:** Thromboelastography: Citrated rapid thromboelastography and citrated functional fibrinogen (mean ± SD).

	Ref. values	LyoPlas	FrontlineODP	OctaplasLG Powder	FFP	Pre‐FrontlineODP	OctaplasLG	*p*‐Value
CRT	*R*, 0.3–1.1 min	0.6 ± 0.1	0.5 ± 0.1	0.5 ± 0.1	0.4 ± 0.1	0.5 ± 0.1	0.5 ± 0.2	ns
	*K*, 0.8–2.7 min	1.0 ± 0.1	1.1 ± 1.0	1.0 ± 0.1	0.9 ± 0.1	0.9 ± 0.1	1.1 ± 0.1	ns
	Angle, 60°–78°	76.2 ± 1.0	74.9 ± 2.0^a^	75.9 ± 0.8	77.0 ± 0.5^a^	76.7 ± 1.7	75.3 ± 1.3	<0.05
	ACT, 82–152 s	103.0 ± 10.5	97.4 ± 9.4	96.1 ± 8.7	85.6 ± 13.8	98.4 ± 7.3	93.8 ± 14.8	ns
	A10, 44–67 mm	60.4 ± 1.8^b^, ^c^	62.3 ± 2.5	62.3 ± 1.3	64.6 ± 1.2^b^	63.9 ± 2.5^c^	62.5 ± 1.8	<0.01
CFF	A10, 15–30 mm	22.6 ± 1.2	23.1 ± 2.6	22.8 ± 0.8	23.6 ± 1.1	23.5 ± 2.3	22.8 ± 1.5	ns

*Note*: *p*‐Value was derived from one‐way ANOVA with Tukey's multiple comparison test.

Abbreviations: A10, amplitude 10 min; ACT, activated clotting time; ANOVA, analysis of variance; FFP, fresh frozen plasma; *K*, clot kinetics; ns, non‐significant; *R*, reaction time.

^a^ FFP in‐house versus FrontlineODP (*p* < 0.05).

^b^ FFP in‐house versus LyoPlas (*p* < 0.01).

^c^ FFP Pre‐FrontlineODP versus LyoPlas (*p* < 0.05).

### Plasma reconstitution and evaluation by potential users

Time measurements including preparation time, time to dissolve and total time showed no statistical difference. Preparation time was around 1.5 min (*p* = 0.977), time to dissolve between 5 and 7.5 min (*p* = 0.062) and total time ranged from 7 to 9 min (*p* = 0.190). However, when comparing the total time using the endpoint of LyoPlas being transferred back to the empty water bag, it took a significantly longer time compared with OctaplasLG Powder (*p* = 0.033) (Table 3).

**TABLE 3:** Reconstitution of dried plasma.

	LyoPlas		OctaplasLG Powder		FrontlineODP	
	Mean	SD	Mean	SD	Mean	SD
Preparation	1 min 37 s	1 min 9 s	1 min 43 s	43 s	1 min 41 s	50 s
Time to dissolve	5 min 35 s	2 min 7 s	4 min 45 s	2 min 29 s	7 min 31 s	2 min 32 s
Total time	7 min 22 s	2 min 31 s	6 min 28 s	3 min 3 s	9 min 12 s	2 min 41 s
Total time to bag	11 min 4 s	3 min 40 s	na		na	

Abbreviation: na, not applicable.

**FIGURE 3:**
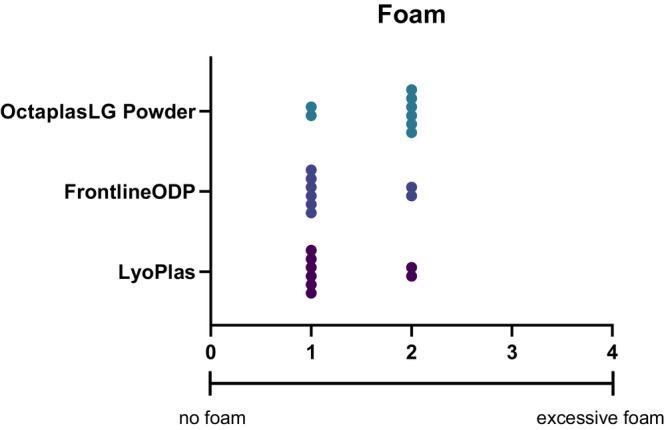
Subjective rating of foam after dissolving each dried plasma. Each dot represents one test user's rating, with a minimum value of 0 (no foam) and a maximum value of 4 (excessive foam).

The test users found quick reconstitution from dried to liquid product to be the most important property of DP. They also had opinions on the DP package in either glass bottles or plastic bags. All users preferred to have the reconstituted plasma in a plastic bag as it makes it possible to transfuse plasma under high pressure. Furthermore, foam in the reconstituted product was assessed. None of the reconstituted DP showed excessive foam (Figure 3), and after transferring the dissolved LyoPlas to the empty plastic bag, no foam was observed.

The subjective opinions from the test users suggest that all the DP products are satisfactory to use, with minor differences in personal preference. One of the test users thought FrontlineODP was more difficult to dissolve in a plastic bag compared with the other two products dissolved in glass bottles. Two users were critical of the use of a needle instead of a spike in the connection device for OctaplasLG Powder, increasing the risk of perforating the water bag or stabbing yourself. The different forms of glass bottles were also commented on, with the OctaplasLG Powder bottle considered clumsier than the LyoPlas bottle.

## DISCUSSION

DP is increasingly requested, and new products are being developed, recently approved or under approval. In this study, we have evaluated three differently produced DP products. The concentration of coagulation factors was within normal human reference values for all products. Still, as expected, more variations were seen in the single‐unit‐prepared LyoPlas and FrontlineODP compared with OctaplasLG Powder made from standardized pools. The coagulation factor content was slightly reduced in the dried products compared with the fresh plasma, which follows previously published data demonstrating a 10%–20% loss depending on the measured factor [6, 8, 9, 10]. Labile factor VIII is the most sensitive and is usually reduced by about 20%–25% after drying [18], also confirmed in our study. vWF is a factor reported to lose activity after freeze drying [10, 18]. However, in this study, the vWF antigen expression was measured, and the antigen levels were well within reference values. The aPTT was prolonged in LyoPlas compared with a study by Bux et al. [10]. This may be due to different methodologies or analysing the product at the end of shelf life. However, Zur et al. have shown a minimal decrease in clotting factors after storage of LyoPlas under field conditions, even beyond the expiration date [2, 9]. INR was lower for OctaplasLG Powder compared with the other two DP products, although INR was within the normal range for all products. Thromboelastography displayed that the clot formation time and clot firmness were comparable among the tested DP. However, LyoPlas and FrontlineODP showed minor changes in CRT A10 and angle compared with fresh products.

The time to reconstitute the DP in sterile water was similar despite the differences in production and packaging, with FrontlineODP in a plastic bag and the other products in glass bottles. For the LyoPlas product, the dissolved plasma could be transferred back to the empty water bag, which was not an option for OctaplasLG Powder. The total reconstitution time that ranged from 7 to 9 min can probably be reduced to less than 5 min for all the products with some practice, supported by the fact that the experienced users in this study could reconstitute the plasma in less than 4 min, with no differences between the products. It was preferred by all users to transfuse the plasma from a plastic bag as it allows them to squeeze the bag and transfuse under pressure, which was possible with both FrontlineODP and LyoPlas, but not for OctaplasLG Powder. The connection tube between the bottle and the water bag in OctaplasLG Powder had a needle in one end, instead of a spike, which was confusing, and with the risk of perforating the water bag or stabbing yourself.

The main difference between the evaluated products is the production process. LyoPlas and OctaplasLG Powder are freeze‐dried through lyophilization, whereas LyoPlas is produced from single‐unit plasma and OctaplasLG Powder from large pools. FrontlineODP is aimed to be prepared at national or local blood centres using a spray‐drying device (FrontlineODP system). Single plasma units are spray‐dried in the instrument, which enables the production volume to be locally or nationally planned and managed [8]. Furthermore, OctaplasLG Powder is pathogen‐reduced with solvent detergent technique, while the other two products are not pathogen‐reduced. A fourth alternative not included in the study is the French lyophilized freeze‐DP (FLYP). FLYP is a pooled (11 donors), ABO‐universal and pathogen‐reduced with amotasalen (INTERCEPT) [5, 11]. FLYP is used by the French and US military and in French civilian care. More than 1100 units have been transfused with no reactions or infectious complications [19, 20].

The different production processes are important when planning the availability, requested volumes and safety of the products. Producing DP from pools allows for more standardized products with less unit‐to‐unit variability. However, pooling can increase the risk of bloodborne disease transmission; therefore, pathogen reduction must be done [21, 22]. The pooling might complicate the production and further limit the availability, for example, if the pooled product consists of only blood group AB [23]. Another aspect is the centralization of DP manufacturing. Centralization promotes good ‘pharmaceutical‐like’ production with more standardized protocols. However, centralization can be vulnerable as production is restricted to one or a few facilities. With a decentralized production, each nation or blood centre can control and regulate production according to its needs. Decentralization also promotes collaborations between centres to cover a larger area [24].

The indications of treatment with DP are currently under discussion and may lead to increased requests, not only by the military but also in civilian care. The military has used DP for decades without major reactions or adverse events [1]. In civilian care, the outcome from plasma studies has been inconclusive, and it is still unclear under which circumstances pre‐hospital transfusion of DP is beneficial [25, 26, 27, 28, 29, 30, 31, 32]. It is supported that in longer transportation time (>20 min) plasma compared with clear fluids may reduce mortality [25, 26, 33]. However, even if the implementation of DP in civilian health care may be uncertain, the logistical advantages of DP will likely lead to increased use as it becomes commonly available.

In conclusion, the three evaluated DP products, LyoPlas, FrontlineODP and OctaplasLG Powder, showed high in vitro quality containing adequate concentrations of coagulation factors and displayed normal clot formation, measured by TEG. These results support that DP can be produced using lyophilization or spray‐drying technique, as well as with the addition of pathogen reduction, with preserved coagulation capability. The products were reconstituted in an acceptable time and deemed feasible by eighth test users, with the reservation that plastic bags were preferred to bottles. DP is logistically superior to fresh plasma with potential widespread availability that may improve bleeding resuscitation. Still, more clinical studies in a pre‐hospital environment are required to understand where and when treatment with DP is indicated and the volumes of DP needed in the civilian health care, as well as in national planning.

### Limitations

This study is an experimental study of three differentially produced DP products measuring the concentration of selected coagulation factors and clot formation in vitro after reconstitution. The results were compared with thawed plasma before drying, but a strictly paired comparison could only be done for FrontlineODP. This discrepancy may be most crucial for LyoPlas as the origin plasma is from single donor with a greater variation than OctaplasLG created in large batches. Subjective opinions from eight users may not be representative of all clinical users.

## CONFLICT OF INTEREST STATEMENT

The authors declare no conflicts of interest.

## Data Availability

The data that support the findings of this study are available from the corresponding author upon reasonable request.
